# Carbapenemase-producing rare Enterobacterales in hospital food: a Latin American one health blind spot

**DOI:** 10.3389/fmicb.2026.1978607

**Published:** 2026-10-07

**Authors:** Bianca L. C. de Godoy, Giovana Rosa, Mara C. L. Nogueira, Nilton Lincopan, Tiago Casella

**Affiliations:** 1 Center for Microorganism Research, Faculdade de Medicina de São José do Rio Preto—FAMERP, São José do Rio Preto, Brazil; 2 Hospital de Base—Funfarme, São José do Rio Preto, Brazil; 3 Department of Microbiology, Institute of Biomedical Sciences, University of São Paulo, São Paulo, Brazil; 4 Antimicrobial Resistance Institute of São Paulo (ARIES), São Paulo, Brazil

**Keywords:** carbapenemase production, critical priority pathogens, genomic surveillance, hospital settings, ready-to-eat food

## Abstract

**Introduction:**

Carbapenemase-producing Enterobacterales (CPE) are a global public health concern. These bacteria are very common in human infections, but reports of their presence in the food chain are increasing and could represent alternative routes for colonization and infection. Hence, we conducted a surveillance study to investigate CPE in food distributed to patients at a tertiary hospital in Brazil.

**Methods:**

The samples were collected right before distribution and screened for β-lactam-resistant Enterobacterales. Isolates that exhibited carbapenem resistance were screened for NDM, IMP, VIM, OXA-48-like, and KPC carbapenemase genes by multiplex polymerase chain reaction (PCR), and those identified as CPE underwent whole-genome sequencing.

**Results:**

Accordingly, we report the detection of a KPC-2-producing *Superficieibacter electus*, a KPC- 2-producing *Pseudocitrobacter faecalis*, and two NDM-1-producing *Leclercia adecarboxylata* isolated from four distinct food samples. The carbapenemase genes were harbored by plasmids, and the genomic traits indicate their potential to cause hard-to-treat human infections or serve as a reservoir of important antimicrobial resistance genes. Moreover, despite the 3-month time span between collections, the two *L. adecarboxylata* isolates exhibited high similarity, suggesting horizontal transmission of CPE associated with the hospital food service.

**Discussion:**

Thus, the emergence of rare Enterobacterales raises concerns about the potential for silently spreading, hard-to-treat infections.

## 1 Introduction

Carbapenem-resistant Enterobacterales remain an urgent public health issue that demands action worldwide (Di Pilato et al., 2024), and the *Klebsiella pneumoniae* carbapenemase (KPC) and the New Delhi Metallo-β-Lactamase (NDM) variants are among the main mechanisms of carbapenem resistance. In addition to human clinical isolates, carbapenemases are also detected in bacteria from environmental and zoonotic sources (Hammoudi Halat and Ayoub Moubareck, 2020) and in contaminated foods (Harding-Crooks et al., 2023), which raises concerns about the need for surveillance and public health measures to control their dissemination.

The rapid spread of carbapenem-resistant pathogens recognized by the World Health Organization (WHO) is a public health problem no longer restricted to hospital settings. It is now regarded as a critical One Health issue (Sati et al., 2025). In this context, in March 2025, the Panel on Biological Hazards (BIOHAZ) of the European Food Safety Authority (EFSA) published a scientific opinion on the identification of carbapenemase-producing Enterobacterales in the food chain across 14 of 30 European Union/European Free Trade Association (EU/EFTA) countries. This report highlighted *Escherichia coli* as the primary focus of the majority of studies, and it identified the *Enterobacter cloacae* complex, *Klebsiella pneumoniae* complex, and *Salmonella* Infantis as the most frequent carbapenemase-producing Enterobacterales (CPE), which carried *bla*_VIM-1_, *bla*_OXA-48_, and *bla*_OXA-181_, *bla*_NDM-5_, and *bla*_IMI-1_ carbapenemase genes (Allende et al., 2025).

In Latin America, KPC-producing Enterobacterales have reached endemic levels in hospital settings, with increasing reports of NDM-producing strains throughout the region. In the Brazilian food chain, KPC-2-producing *K. pneumoniae* has been sporadically reported in poultry in the Brazilian Amazon Region (Valiatti et al., 2022). However, the increasing detection of international high-risk clones of *E. coli*, *K. pneumoniae* complex, *K. michiganensis*, *Enterobacter hormaechei*, and *Pseudomonas aeruginosa* producing KPC-2, NDM-1, or VIM-2 carbapenemases in fresh and ready-to-eat vegetables in the country (Furlan et al., 2024; Godoy et al., 2024; Venâncio de Godoy et al., 2024; Dantas et al., 2025) represents a critical food safety concern. These findings suggest that these pathogens may be transmitted to humans through the food chain (Furlan et al., 2024; Godoy et al., 2024; Venâncio de Godoy et al., 2024; Allende et al., 2025; Dantas et al., 2025). Therefore, we conducted a surveillance program in a tertiary-care hospital in southeastern Brazil to investigate the occurrence of WHO-recognized carbapenem-resistant pathogens in food samples collected from the hospital food service. In this study, we report the identification of four rare carbapenem-resistant Enterobacterales recovered from meat-based and vegetarian meals.

## 2 Materials and methods

### 2.1 Sample collection, bacterial isolation, and pH tolerance

From May 2022 to April 2023, we conducted an observational, descriptive, serial cross-sectional study to investigate the burden of antimicrobial resistance in food distributed to inpatients at a tertiary-care teaching hospital in São Paulo, Brazil. During this surveillance, 191 food samples (beef, pork, or chicken meat preparations and raw salads) were collected from the hospital food service right before the distribution of meals to the patients in a sterile 500-mL plastic bag and transported to the laboratory at room temperature (which matched the temperature at which the meals were served to patients) within 15 min for the following procedures.

In summary, approximately 25 g of each sample was aseptically weighed, enriched with 225 mL of peptone water (Oxoid, UK) supplemented with 2 mg/L of ceftriaxone, and incubated overnight at 35 ± 2 °C. Next, a 10 μL aliquot was plated onto MacConkey agar plates (MC; Oxoid, UK) supplemented with 2 mg/L of ceftriaxone (CRO) and further incubated overnight at 35 ± 2 °C. Colonies presenting different morphologies characteristic of Enterobacterales were isolated for use in this study (Venâncio de Godoy et al., 2024). Bacterial identification was performed using the matrix-assisted laser desorption/ionization time-of-flight mass spectrometry (VITEK® MS PRIME System, France) method.

The tolerance of isolates to different pH values was tested in trypticase soy broth (Oxoid, UK) culture medium using 96-well polystyrene plates to mimic conditions of the gastrointestinal tract (pH 2.0–9.0) after 1 h, 2 h, and 24 h of incubation. The assay was performed following the methodology described in a previous publication (Lopes et al., 2021), with minor modifications.

### 2.2 Antimicrobial susceptibility testing

Antimicrobial susceptibility was determined using the disk diffusion method on Müeller-Hinton agar (Oxoid, UK), following the guidelines established by the Brazilian Committee on Antimicrobial Susceptibility Testing (BrCAST, 2024). The following antimicrobials were tested: amoxicillin/clavulanic acid, piperacillin/tazobactam, cefoxitin, ceftriaxone, cefepime, ertapenem, amikacin, gentamicin, ciprofloxacin, tetracycline, trimethoprim/sulfamethoxazole, and chloramphenicol. The minimal inhibitory concentration for polymyxin B was determined by a commercial broth microdilution method (Policimbac®, Probac, Brazil). The interpretative criteria were based on the BrCAST/EUCAST guidelines (BrCAST, 2024). *E. coli* ATCC 25922 and *K. pneumoniae* ATCC 700603 strains were used as controls for the antimicrobial susceptibility tests.

Carbapenemase production was screened using the modified carbapenem inactivation method (mCIM) and the EDTA-carbapenem inactivation method (eCIM) (CLSI, 2018), and detection of the most prevalent carbapenemase families (i.e., NDM, IMP, VIM, OXA-48, and KPC) in phenotypically positive strains was performed using multiplex PCR, as previously described (Doyle et al., 2012). Multidrug-resistant (MDR) bacteria are defined as those that show resistance to at least one antimicrobial agent in at least three drug classes (Magiorakos et al., 2012).

### 2.3 Plasmid characterization

Plasmids were classified using the replicon typing scheme with the commercial PBRT-2 kit (Diatheva, Italy). Pulsed-field gel electrophoresis after S1 enzyme (Probac, Brazil) digestion (PFGE-S1), followed by Southern blot, was performed electronically using the Power Blotter Semi-dry Transfer System (Thermo Fisher Scientific, United States). The subsequent hybridization was performed using adequate probes and reagents (Roche) to verify plasmid sizes and types (Schultz et al., 2017).

### 2.4 Whole-genome sequencing and genomic analysis

Genomic DNA of the rare Enterobacterales was extracted using the PureLink™ Genomic DNA Mini Kit (Thermo Fisher Scientific, United States), and the concentration was assessed using the Qubit dsDNA High Sensitivity Assay Kit (Thermo Fisher Scientific, United States). The sequencing library was prepared using the Illumina COVIDSeq® Assay (Illumina, Inc., CA, United States) for 150-bp paired-end reads, following the manufacturer’s instructions, and the whole genome was sequenced on an Illumina MiSeq platform (Illumina, United States) with a MiSeq® Reagent Kit v3 (600-cycle). Trimmomatic v0.39 (Bolger et al., 2014) and SPAdes v4.0.0 (Bankevich et al., 2012) were used to trim the reads and perform *de novo* assembly.

The scaffolds were submitted to the Type Strain Genome Server^1^ and the fIDBAC platform^2^ for species identification using the average nucleotide identity (ANI) calculator. Antimicrobial resistance genes, plasmid replicon types, virulome, and the probability of being a human pathogen were assessed using ResFinder 4.7.2 (Bortolaia et al., 2020), PlasmidFinder 2.1 (Carattoli et al., 2014), VFanalyzer (Liu et al., 2019), and PathogenFinder 2 (Ferrer Florensa et al., 2026), respectively.

Isolates identified as *Leclercia adecarboxylata* were compared with others isolated worldwide. For this purpose, a phylogenetic tree was inferred based on the concatenated alignment of the single-nucleotide polymorphism (SNP) sequences of the isolates obtained in this study and all 24 complete genomes identified as *L. adecarboxylata* available at the National Center for Biotechnology Information (NCBI) on 25 June 2026, using the default parameters of CSI Phylogeny 1.4 (Kaas et al., 2014). The assembled genome corresponding to NCBI accession ASM2009739v1 was used as a reference and included in the tree. The resulting tree was visualized and pruned on Interactive Tree Of Life (iTOL) v6 (Letunic and Bork, 2024).

## 3 Results

### 3.1 Bacterial detection, pH tolerance, antimicrobial resistance, and carbapenemase genes

During a year of monitoring local antimicrobial resistance in hospital food, an average of 15.9 samples were tested weekly, resulting in 79 Enterobacterales isolates, of which 10 (12.7%) were identified as CPE. Some of these CPE were already described by us (Godoy et al., 2024; Venâncio de Godoy et al., 2024), and four other CPE isolates (HB23, HV46, HV56B, and HV65B) were identified as species rarely associated with human infections. These were isolated from four distinct salad or meat preparations: *Superficieibacter electus* (strain HB23), recovered from a beef preparation meal on 19 October 2022; *Pseudocitrobacter faecalis* (strain HV46), obtained from a cabbage salad on 13 December 2022; and two *Leclercia adecarboxylata* (strains HV56B and HV65B), collected from cabbage and lettuce salads on 25 January 2023 and 12 April 2023, respectively. All four isolates presented tolerance to pH values 4.0–9.0 for up to 24 h, and HV46 also tolerated pH 3 for at least 1 h.

The four uncommon isolates showed resistance to all tested *β*-lactams, while resistance to the other antimicrobial classes tested varied (Table 1). Due to ertapenem resistance and the profiles of other β-lactams, a phenotypic screening method for carbapenemase production was applied, which revealed serine carbapenemase production by HB23 and HV46 and metallo-β-lactamase production by HV56B and HV65B.

**Table 1:** Antimicrobial susceptibility profile of *S. electus* HB23, *P. faecalis* HV46, *L. adecarboxylata* HV56B, and *L. adecarboxylata* HV65B.

Antimicrobials	Susceptibility profile^a^			
	HB23	HV46	HV56B	HV65B
Amoxicillin-clavulanic acid	R	R	R	R
Piperacillin-tazobactam	R	R	R	R
Cefoxitin	R	R	R	R
Ceftriaxone	R	R	R	R
Cefepime	R	R	R	R
Ertapenem	R	R	R	R
Amikacin	S	S	R	S
Gentamicin	S	S	S	R
Ciprofloxacin	S	S	R	R
Tetracycline	S	S	R	R
Trimethoprim-sulfamethoxazole	S	S	R	R
Chloramphenicol	S	S	S	S
Polymyxin B	S	S	S	S

^a^Susceptibility profile of bacteria was determined by the disk-diffusion method, with exception for Polymyxin B that was determined by microdilution method. The interpretative criterion was based on the BrCAST/EUCAST guidelines (BrCAST, 2024). *R: resistant; S: susceptible.

The genotypic screening of carbapenemase genes revealed the presence of *bla*_KPC_ in *S. electus* HB23 and *P. faecalis* HV46, and *bla*_NDM_ in the two *L. adecarboxylata* HV56B and HV65B. The plasmid replicon typing and PFGE-S1 experiments showed that HB23 and HV46 carried the *bla*_KPC_ gene harbored by an IncN2 plasmid, which is approximately 85 and 90 kb, respectively, while HV56B and HV65B presented the *bla*_NDM_ harbored by an IncC plasmid, with sizes of approximately 225 and 235 kbp, respectively.

### 3.2 Genomic analysis

The four rare isolates underwent whole-genome sequencing (WGS) for further characterization (Table 2). The species were confirmed as previously identified, and the strains *S. electus* HB23 and *P. faecalis* HV46 carried the *bla*_KPC-2_ allele of the gene, and both *L. adecarboxylata* strains HV56B and HV65B carried the allele *bla*_NDM-1_. Moreover, the resistome analysis revealed that HB23 and HV46 further carried only the *qnr*VC1 gene that expresses resistance to quinolones, while HV56B and HV65B carried additional genes encoding resistance to β-lactams (*bla*_CARB-2_, *bla*_OXA-9_, *bla*_TEM-1A_), aminoglycosides (*aac*(6′)-Ib, *aad*A1, *aph*(3′)-VI, *aph*(3″)-Ib, *aph*(6)-Id), quinolones (*qnr*B19), sulphonamides (*sul*1, *sul*2), trimethoprim (*dfr*A23), fosfomycin (*fos*A), tetracyclines [*tet*(A)], and quaternary ammonium compounds (*qac*EΔ1).

**Table 2:** Genomic characteristics of *S. electus* HB23, *P. faecalis* HV46, *L. adecarboxylata* HV56B, and *L. adecarboxylata* HV65B.

Characteristic	*S. electus* HB23	*P. faecalis* HV46	*L. adecarboxylata* HV56B	*L. adecarboxylata* HV65B
Resistome	*qnr*VC1, *bla*_KPC-2_	*qnr*VC1, *bla*_KPC-2_	*aac*(6′)-Ib, *aad*A1, *aph*(3′)-VI, *aph*(3″)-Ib, *aph*(6)-Id, *qnr*B19, *bla*_CARB-2_, *bla*_NDM-1_, *bla*_OXA-9_, *bla*_TEM-1A_, *sul*1, *sul*2, *dfr*A23, *fos*A, *tet*(A), *qac*E	*aac*(6′)-Ib, *aad*A1, *aph*(3′)-VI, *aph*(3″)-Ib, *aph*(6)-Id, *qnr*B19, *bla*_CARB-2_, *bla*_NDM-1_, *bla*_OXA-9_, *bla*_TEM-1A_, *sul*1, *sul*2, *dfr*A23, *fos*A, *tet*(A), *qac*E
Plasmidome	IncN2, IncX10	Col(IRGK), Col(pHAD28), Col440I, IncHI1A, IncHI1B, IncN2, IncR, pKP1433	Col(pHAD28), IncC, IncR	Col(pHAD28), IncC, IncR
Virulome	ACE T6SS, *acr*AB, *all*B, Capsule, *che*B, *che*W, *che*Y, Ent siderophore, *flg*C, *flg*G, *flg*H, *flh*A, *flh*C, *fli*A, *fli*G, *fli*I, *fli*M, *fli*N, *fli*Q, *fli*Y, *fep*C, *fur*, *gal*F, *mot*A, *nlp*I, *rcs*AB, *rpo*S, *sit*C, T6SS-II	ACE T6SS, *acr*AB, *asl*A, Capsule, *che*B, *che*W, *che*Y, *clp*B, *clp*V, *flg*C, *flg*G, *flg*H, *flh*A, *flh*C, *fli*A, *fli*G, *fli*I, *fli*M, *fli*N, *fli*Q, *fur*, *gal*F, *ipa*H, *iut*A, *mot*A, *nlp*I, *omp*A, *pho*P, *ppd*D, *rcs*AB, *rpo*S, Type 3 fimbriae, *vgr*G	*acr*AB, Capsule, *che*B, *che*R, *che*W, *che*Y, *che*Z, *csg*G, Ent siderophore, *flg*C, *flg*G, *flg*H, *flg*I, *flh*A, *flh*C, *fli*A, *fli*G, *fli*I, *fli*M, *fli*N, *fli*P, *fli*Y, *fur*, *mot*A, *nlp*I, *pho*P, *rcs*AB, *rpo*S, T6SS-I, T6SS-II, *ugd*	*acr*AB, Capsule, *che*B, *che*R, *che*W, *che*Y, *che*Z, *csg*G, Ent siderophore, *flg*C, *flg*G, *flg*H, *flg*I, *flh*A, *flh*C, *fli*A, *fli*G, *fli*I, *fli*M, *fli*N, *fli*P, *fli*Y, *fur*, *mot*A, *nlp*I, *pho*P, *rcs*AB, *rpo*S, T6SS-I, T6SS-II, *ugd*
Human pathogenicity prediction score (mean)	0.9454	0.9712	0.9712	0.9736
GenBank BioSample accession numbers	SAMN40726037	SAMN40726038	SAMN40726039	SAMN40726040

Regarding the plasmidome, HB23, HV56B, and HV65B harbored only a few plasmids, while numerous replicon types were identified in HV46. With respect to the pathogenicity traits, all four genomes were predicted as probable human pathogens. According to the PathogenFinder 2 tool, pathogenicity proteins of HB23 was closely related to a *Pantoea eucrina* (BioSample SAMN05757669) isolated in 1992 from a human in the US; the proteins of HV46 were closely related to an *E. coli* (BioSample SAMD00077531) isolated in 2014 from cattle feces in Japan; the proteins of HV56B were closely related to a *Citrobacter freundii* (BioSample SAMN17372996) isolated in 2019 from a human rectal swab in Germany; and the protein set of HV65B was related to an *E. coli* (BioSample SAMN05283707) isolated in 2012 from human urine in Denmark.

The four genomes also contained virulence genes for regulation, type-6 secretion systems, peritrichous flagella, capsules for immune evasion and antiphagocytosis, efflux pumps, and iron acquisition. However, genes for allantoin utilization were additionally predicted in the *S. electus* HB23 genome, those encoding the type 3 fimbriae, invasion, Mxi-Spa type-3 secretion system effectors, and the hemorrhagic *E. coli* pilus (HCP) in the genome of *P. faecalis* HV46, and genes for Curli fiber expression in the *L. adecarboxylata* HV56B and HV65B genomes.

### 3.3 Phylogenomic analysis of *L. adecarboxylata* worldwide

We used the 24 *L. adecarboxylata* complete genomes publicly available at the time of this submission for comparison with our two isolates, as depicted in Table 3. The available information on those genomes revealed that the respective strains were mainly isolated from China (*n* = 14), especially from human and environmental samples from 2012 to 2025.

**Table 3:** Epidemiological and genomic data on the two *L. adecarboxylata* HV56B and HV65B isolated in this study and on the 24 publicly available complete genomes of *L. adecarboxylata* available in the NCBI repository (https://www.ncbi.nlm.nih.gov/datasets/genome/).

RefSeq accession	Host/Source	Sample type	Collection date	Geographic location	Pathogen prediction	Antimicrobial resistance genes
GCF_001518835.1	Calf	Nasopharyngeal swab	2013	USA	0.9641	—
GCF_004295325.1	*Homo sapiens*	Sputum	2016	China	0.9652	—
GCF_006171285.1	*Homo sapiens*	Stool	2018	China	0.9693	*qnr*S1, *bla*_IMP-4_, *mcr*-4.3
GCF_006874705.1	Rabbit	Unknown	2015	China	0.9624	*aac*(6′)-Ib-cr, *aad*A16, *sul*1, *dfr*A27, *flo*R, *ARR*-3, *qac*E
GCF_008807335.1	*Homo sapiens*	Secretion (osteomyelitis)	2018	China	0.9631	*mcr*-9.1
GCF_008931385.1	Environmental	Shower 2	2014	Australia	0.9681	*aac*(3)-IId, *aac*(6′)-Ib3, *qnr*B2, *bla*_TEM-1B_, *bla*_IMP-4_, *sul*1, *mph*(A), *cat*B3, *mcr*-9.1, *form*A, *qac*E
GCF_008931445.1	Environmental	Shower 3	2012	Australia	0.9748	*aac*(3)-IId, *aac*(6′)-Ib3, *bla*_TEM-1B_, *bla*_IMP-4_, *sul*1, *mph*(A), *cat*B3, *mcr*-9.1, *form*A, *qac*E
GCF_009720165.1	*Homo sapiens*	Drainage fluid (gastric cancer)	2013	China	0.9698	—
GCF_011045715.1	*Homo sapiens*	Bile	2018	China	0.9632	*mcr*-9.1
GCF_011045735.1	*Homo sapiens*	Blood	2017	China	0.9616	*arm*A, *qnr*B4, *bla*_DHA-1_, *bla*_SHV-12_, *sul*1, *msr*(E), *mph*(E), *mcr*-9.1, *qac*E
GCF_011387015.1	*Homo sapiens*	Blood	2015	China	0.9678	—
**GCF_020097395.1**	Unknown	Unknown	Unknown	Germany	0.9677	—
GCF_020990425.1	Food	Pig feed	2019	China	0.9773	*aac*(6′)-Ib-cr, *aph*(6)-Id, *aph*(3″)-Ib, *aph*(3′)-Ia, *qnr*S1, *bla*_OXA-1_, *bla*_LAP-2_, *sul*1, *dfr*A14, *cfr*, *msr*(E), *mph*(A), *mph*(E), *tet*(A), *flo*R, *cat*B3, *ARR*-3, *qac*E
GCF_023612295.1	Environmental	Garlic rhizosphere soil	2019	China	0.9689	—
GCF_023639785.1	*Sesuvium verrucosum*	Root sample	2020	United Arab Emirates	0.9609	—
GCF_025790845.1	*Homo sapiens*	Rectal Swab	2022	USA	0.9797	*aac*(6′)-Ib3, *aph*(6)-Id, *aph*(3″)-Ib, *aad*A1, *aph*(3′)-XV, *qnr*S1, *bla*_SHV-12_, *bla*_VIM-1_, *sul*1, *sul*2, *dfr*A14, *mph*(A), *cat*B2, *mcr*-9.1, *qac*E
GCF_030388365.1	*Homo sapiens*	Stool	2017	China	0.9651	*aac*(6′)-Ib3, *aad*A1, *aad*A6, *aph*(3′)-Ia, *qnr*B19, *bla*_IMP-1b_, *sul*1, *dfr*A1, *tet*(A), *qac*E
GCF_032598765.1	Environmental	Biofertilizer	Unknown	Malaysia	0.9683	*aph*(6)-Id, *aph*(3″)-Ib, *bla*_TEM-1B_, *sul*2, *dfr*A14, *tet*(D), *cat*A2, *mcr*-9.1, *form*A
GCF_036771045.1	Environmental	Rhizosphere soil of maize	2019	China	0.9712	*aad*A1, *aph*(3′)-Ia, *qnr*B19, *sul*1, *dfr*A1, *tet*(A), *tet*(D), *qac*E
GCF_039085625.1	Environmental	Wastewater	2023	South Korea	0.9708	*aac*(3)-IId, *aph*(6)-Id, *aad*A22, *qnr*S13, *bla*_CTX-M-55_, *sul*3, *dfr*A14, *lnu*(F), *mph*(A), *tet*(A), *mcr*-9.1, *ARR*-2
GCF_041725765.1	Environmental	Corn rhizosphere soil	2022	China	0.9773	*aad*A1, *aad*A2b, *aph*(3′)-Ia, *qnr*B19, *sul*1, *dfr*A1, *tet*(A), *tet*(D), *cat*A2, *mcr*-9.1, *qac*E
GCF_054796225.1	*Homo sapiens*	Unknown	2025	China	0.9745	*aac*(6′)-Ib3, *qnr*S1, *bla*_CTX-M-3_, *bla*_TEM-1B_, *bla*_OXA-1_, *bla*_NDM-1_, *fos*A, *cat*B3, *sul*1, *ARR*-3
GCF_056155235.1	Environmental	Soil surface	2022	Switzerland	0.9705	*aph*(3′)-Ia, *aad*A2b, *bla*_OXA-48_, *bla*_TEM-1B_, *cat*A1, *sul*1, *tet*(B)
GCF_901472455.1	Food	Drinking water	Unknown	Unknown	0.8799	—
SAMN40726039*	Food	Cabbage	2023	Brazil	0.9712	*aac*(6′)-Ib, *aad*A1, *aph*(3′)-VI, *aph*(3″)-Ib, *aph*(6)-Id, *qnr*B19, *bla*_CARB-2_, *bla*_NDM-1_, *bla*_OXA-9_, *bla*_TEM-1A_, *sul*1, *sul*2, *dfr*A23, *fos*A, *tet*(A), *qac*E
SAMN40726040*	Food	Lettuce	2023	Brazil	0.9736	*aac*(6′)-Ib, *aad*A1, *aph*(3′)-VI, *aph*(3″)-Ib, *aph*(6)-Id, *qnr*B19, *bla*_CARB-2_, *bla*_NDM-1_, *bla*_OXA-9_, *bla*_TEM-1A_, *sul*1, *sul*2, *dfr*A23, *fos*A, *tet*(A), *qac*E

*The last two genome rows denote the strains HV56B and HV65B sequenced in this study, respectively, and are referred to by the BioSample accession numbers.

All the information on the deposited genomes was directly collected from the NCBI database as reported by the respective submitter. The RefSeq accession of the genome used as the reference strain (as established by the NCBI team) is bolded.

Regarding the probability of being a human pathogen, the mean of all genomes was 0.9656, ranging from 0.8799 (an isolate obtained from drinking water) to 0.9797 (an isolate obtained from a human rectal swab), which demonstrates that this species has a high predicted probability of causing human infection.

With respect to the resistome, only one of these genomes (NCBI RefSeq accession GCF_054796225.1) carried the *bla*_NDM-1_ gene, but six (25.0%) additional genomes also carried one carbapenemase gene (*bla*_IMP-1b_, *bla*_IMP-4_, *bla*_OXA-48_, or *bla*_VIM-1_). Moreover, colistin-resistance genes (*mcr-4.3* and *mcr-9.1*) were detected in 10 (41.7%) genomes, and in 8 (33.3%) other genomes, no antimicrobial resistance gene was predicted by the ResFinder tool. Genes encoding resistance to aminoglycosides, amphenicols, formaldehyde, fluoroquinolones, lincosamides, macrolides, oxazolidinones, pleuromutilins, quaternary ammonium compounds, rifampicin, streptogramins, sulfamethoxazole, tetracyclines, and trimethoprim were further detected.

A comparative phylogenetic tree was constructed based on SNPs using the whole-genome alignment. SNP counts among strains ranged from 2 to 64,124, and the phylogenetic tree demonstrated pronounced dissimilarity among genomes (Figure 1). The exceptions were the two maize rhizosphere soil isolates collected in China in 2019 and 2022, which differed by only 2 SNPs and some additional antimicrobial resistance genes in the newer isolate, and the two salad isolates (HV56B and HV65B) obtained in this study, which differed by 9 SNPs (Figure 1, Table 3), indicating that the isolates were very closely related to each other.

**Figure 1:**
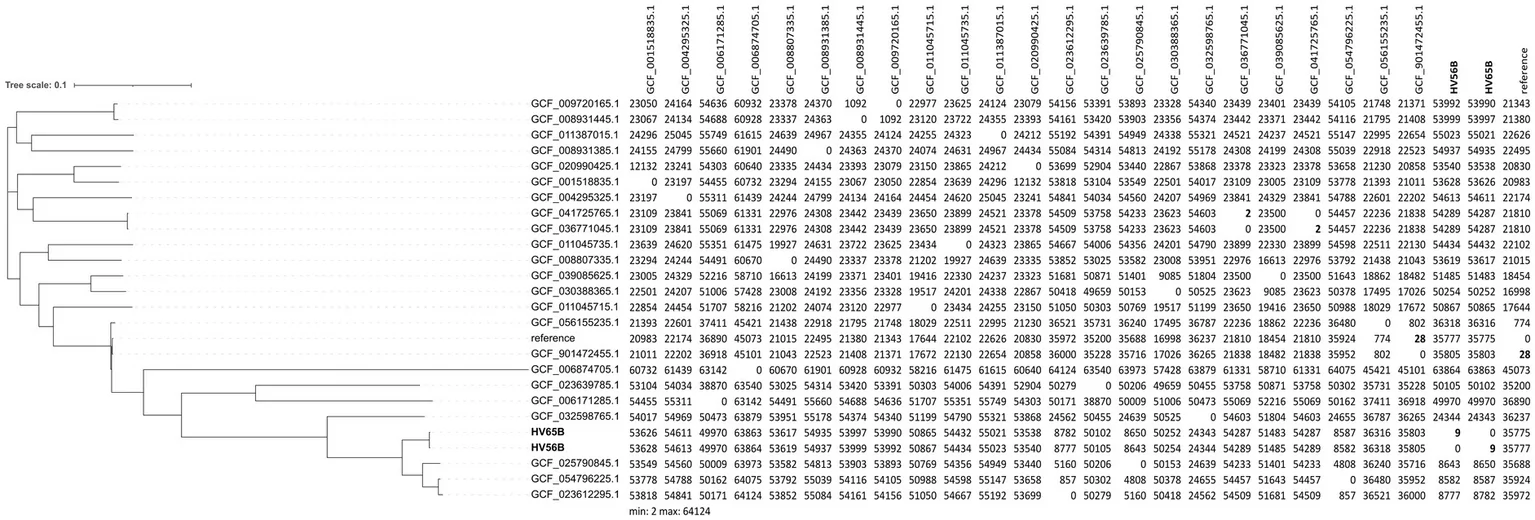
Phylogenetic tree of the 24 publicly available complete genomes at NCBI from *Leclercia adecarboxylata* and the two sequenced in this study. The reference genome used to create the tree was the same one considered the “reference genome” by the NCBI team (NCBI RefSeq accession GCF_020097395.1) and is depicted in the final tree. Genomes are labeled with the respective NCBI RefSeq accession. Strains in bold type are those obtained in this study. The tree scale bar represents the rate of substitutions per site. The SNP tree is followed by the SNP matrix that was used to construct the tree to support the interpretation. Numbers in bold represent up to 30 SNP differences between genomes.

## 4 Discussion

The results presented here support the presence of CPE in food intended for inpatients. Other salads or meat-based meals collected from the institution’s food service were also positive for CPE; the majority of them have already been published (Godoy et al., 2024; Venâncio de Godoy et al., 2024). This is especially significant given that the bacterial species are often neglected as human pathogens but act as reservoirs for carbapenem-resistance genes, potentially enabling horizontal gene transfer to bacteria that colonize or infect hospitalized patients.

Although food contamination can occur at the later stages of the food chain, such as processing and retail, primary production has been considered a more significant contributor to antimicrobial resistance-associated contamination (Harding-Crooks et al., 2023). Therefore, to assess food safety and microbiological quality, in addition to estimating total coliform and *E. coli* counts as indicators of unfavorable hygienic conditions and fecal contamination, the absence of WHO critical-priority Enterobacterales should be used as a microbiological standard (Fuga et al., 2025).

In this respect, the EFSA and the European Centre for Disease Prevention and Control (ECDC) actively monitor the occurrence and spread of CPE in the food chain (Allende et al., 2025). In fact, in some countries, routine monitoring of indicator *E. coli* and *Salmonella* spp., as well as the specific monitoring of extended-spectrum β-lactamase (ESBL)-/AmpC−/carbapenemase-producing *E. coli* in cecal samples of food-producing animals and their meat products, is now mandatory, using culture-based or WGS methods (Allende et al., 2025).

The genomic analysis of the four CPE, rarely identified in human infections, together with phenotypic assays, demonstrated that these bacteria have the potential to overcome the gastrointestinal protective conditions and initiate an infectious process in individuals who ingest them with food. The resistome partly supports the antimicrobial susceptibility profiles, since fosfomycin or quaternary ammonium compounds were not tested to determine resistance in the *L. adecarboxylata* HV56B and HV65B isolates. Moreover, *S. electus* HB23 and *P. faecalis* HV46 presented susceptibility to ciprofloxacin despite the presence of *qnr*VC1. In fact, this gene was described in a ciprofloxacin-resistant *Vibrio cholerae* isolated in the Brazilian Amazon region (Fonseca et al., 2008); however, its presence does not necessarily confer ciprofloxacin resistance (Zhou et al., 2023).

Regarding the potential to cause infection, although these species are considered opportunistic pathogens, the diversity of virulence genes and the high predicted probabilities of being a human pathogen are alarming. Some publications illustrate the association of the three species with humans. The first report of *S. electus* describes the isolation of ESBL-producing isolates from intensive care unit surfaces in Pakistan (Potter et al., 2018). Very few reports describe the isolation of this species. Thus, to the best of our knowledge, this study is the first report of a *bla*_KPC-2_-carrying *S. electus*. On the other hand, carbapenem-resistant *P. faecalis* (Shi et al., 2022) and *L. adecarboxylata* (Yescas-Zazueta et al., 2024) have already been reported in cases of human infections.

Specifically for *L. adecarboxylata*, the general dissimilarities among genomes shown in the SNP tree reinforce the sporadic detection of such pathogens, especially those harboring carbapenemase genes. However, the high similarity observed between the two isolates obtained in this study (fewer than 10 SNPs) strongly suggests a common source for both HV56B and HV65B. We could only speculate on the reason why such similar isolates were detected in different salad samples collected over 3 months from the hospital food service; however, the study was not designed to answer this specific question. Notably, an outbreak associated with total parenteral nutrition contaminated with NDM-producing *L. adecarboxylata* has been reported in three hospitals in western Mexico in 2019 (Garza-González et al., 2021), and the isolation of this organism colonizing a healthy Chinese newborn was also reported (Meng et al., 2023), which demonstrates its contact with immunocompromised patients and its ability to occasionally cause infection.

In summary, the presence of potentially pathogenic, carbapenemase-producing rare Enterobacterales in hospital-ready-to-eat food is concerning. This suggests that food may serve as an unconventional source of antimicrobial resistance genes, potentially leading to hard-to-treat infections through patient colonization. Finally, although foodborne transmission of carbapenemase-producing Enterobacterales is still being characterized, the contamination of food-producing animals and the food chain creates a dangerous reservoir for antibiotic-resistant bacteria and their resistance genes. Therefore, a One Health approach, involving persistent monitoring and research, is critical to protecting public health from these pathogens.

## Data Availability

The datasets presented in this study can be found in online repositories. The names of the repository/repositories and accession number(s) can be found in the article/supplementary material.

## Footnotes

1
https://tygs.dsmz.de/

2
http://fbac.dmicrobe.cn/home/
